# Revitalization of Soil Contaminated by Petroleum Products Using Materials That Improve the Physicochemical and Biochemical Properties of the Soil

**DOI:** 10.3390/molecules29245838

**Published:** 2024-12-11

**Authors:** Jadwiga Wyszkowska, Agata Borowik, Magdalena Zaborowska, Jan Kucharski

**Affiliations:** Department of Soil Science and Microbiology, Faculty of Agriculture and Forestry, University of Warmia and Mazury in Olsztyn, 10-719 Olsztyn, Poland; agata.borowik@uwm.edu.pl (A.B.); m.zaborowska@uwm.edu.pl (M.Z.); jan.kucharski@uwm.edu.pl (J.K.)

**Keywords:** soil contamination, sorbents, soils reclamation, soil quality, oxidoreductases, hydrolases

## Abstract

One of the key challenges in environmental protection is the reclamation of soils degraded by organic pollutants. Effective revitalization of such soils can contribute to improving the climate and the quality of feed and food, mainly by eliminating harmful substances from the food chain and by cultivating plants for energy purposes. To this end, research was carried out using two sorbents, vermiculite and agrobasalt, to detoxify soils contaminated with diesel oil and unleaded gasoline, using maize as an energy crop. The research was carried out in a pot experiment. The level of soil contamination with petroleum products was set at 8 cm^3^ and 16 cm^3^ kg^−1^ d.m. of soil, and the dose of the revitalizing substances, i.e., vermiculite and agrobasalt, was set at 10 g kg^−1^ of soil. Their effect was compared with uncontaminated soil and soil without sorbents. The obtained research results prove that both diesel oil and gasoline disrupt the growth and development of *Zea mays*. Diesel oil destabilized plant development more than gasoline. Both products distorted the activity of soil oxidoreductases and hydrolases, with diesel oil stimulating and gasoline inhibiting. The applied sorbents proved to be useful in the soil revitalization process, as they reduced the negative effects of pollutants on *Zea mays*, increased the activity of soil enzymes, enhanced the value of the biochemical soil quality indicator (BA), and improved the cation exchange capacity (CEC), the sum of exchangeable base cations (EBC), pH, and the C_org_ content. Agrobasalt demonstrated a greater potential for improving soil physicochemical properties, inducing an average increase in CEC and EBC values of 12% and 23%, respectively, in soil under G pressure, and by 16% and 25% in DO-contaminated soil.

## 1. Introduction

Intensive industrialization poses the threat of environmental pollution by various pollutants [1,2]. Crude oil derivatives are particularly hazardous due to their chemical composition and bioavailability [3,4,5]. These products can be transported over long distances, leading to their global dissemination [6] and consequently resulting in their harmful effects on a larger environmental area than would be expected from their localized action [7]. They infiltrate the soil as a result of accidents, disasters, and often with waste and effluents [8,9]. Under natural conditions, they are removed from the soil by biotic and abiotic processes [10]. Biotic processes refer to natural phenomena associated with living organisms and their interactions with each other and the environment, while abiotic processes are associated with physical, chemical, geological, and climatic processes that significantly influence the growth and proliferation of organisms [11]. The metabolism of petroleum products by bacteria is a function of specific genes and enzymes involved in metabolic pathways that degrade aliphatic and aromatic polycyclic hydrocarbons (PAHs) [12]. Goma-Tchimbakala et al. [13] reported the soil microbial diversity in soils under gasoline and diesel oil pressure based on 16 classes and 50 families. The classes are most abundantly represented by *Actinobacteria* and *Gammaproteobacteria*, while the families are represented by *Gordoniaceae* (27%) and *Pseudomonadaceae* (58%). In turn, at the genus level, *Gordonia* (27%) and *Pseudomonas* (58%) were identified. Similarly, according to Wang et al. [14], bacterial genera such as *Pseudomonas* as well as *Bacillus*, *Acinetobacter*, *Sphingobium*, and *Rhodococcus* demonstrate significant biodegradation potential for petroleum compounds. Abiotic factors have a significant influence on the derivatives and end products of PAH degradation. Petrochemical pollutants can impede the movement of water and oxygen in the soil, alter pH, and restrict the availability of nutrients [15,16,17].

The choice of an appropriate soil remediation technique depends on the specific environmental conditions, the type and load of pollutants, and the available financial resources [18,19,20]. Several methods are employed to remove petroleum hydrocarbons [21,22]. The primary method of eliminating these xenobiotics is their transformation [23]. PAHs are persistent compounds, and the process of microbial degradation can be slow and incomplete [16]. Hydrocarbon bioremediation can be significantly enhanced by the use of plants (biologically enhanced phytoremediation) [15,24] and through the application of adsorbents [25,26]. The advantage of adsorbents lies in their low cost and relatively high efficiency in removing contaminants from soil and water [27,28].

The use of adsorbent materials, possessing the ability to bind and retain pollutants on their surface, according to El Mahboubya et al. [27], Singha and Singha [17], Zhang et al. [29], Liu et al. [30], Bandura et al. [31], is effective in reducing the bioavailability of toxic compounds of petroleum-derived substances in the soil. Sorption methods involve the use of adsorbents, which are porous solid bodies with a developed specific surface area capable of binding molecules (from the liquid or gas phase) to their surface [31].

The selected vermiculite (fraction 3–6 mm) chosen for the study is a natural mineral rich in feldspar, quartz, and iron oxides [32]. According to Almuqrin et al. [32], it contains 7% carbonates, 6% feldspars, 23% quartz, and about 17% iron oxides. Vermiculite is characterized by a unique layered structure [33,34], which makes it an excellent adsorption material [32,35]. Its lightweight structure consists of layers, between which water (5–20%) is present, together with exchangeable cations. Vermiculite possesses a negative charge, which allows it to exchange cations. Its structure, composition, surface characteristics, and chemical and physical properties allow the adsorption of molecules on the surface and in the interlayer spaces, making it suitable for soil remediation with good efficacy [36,37,38].

Agrobasalt can be classified as a soil conditioner. Its structure is based on a rock matrix [39]. It is a natural clay–silicate material that is readily available, non-toxic, and inexpensive. The primary minerals of natural basalt include olivine, plagioclase, pyroxene, Fe–Ti oxide, and apatite, while the secondary minerals may include quartz, calcite, clinochlore, sericite, prehnite, pumpellyite, and muscovite [40,41]. It may occur in the form of granules (fraction 1–6.3 mm). Like vermiculite, it is characterized by high water and cation exchange capacities. Thus, it can act as an ion carrier in ion exchange processes, where ions from the solution are adsorbed on the mineral surface and then released into the solution in the exchange process. The sorption properties of agrobasalt are due to its porous structure and ability to adsorb chemicals onto the mineral surface [42,43]. Due to its high specific surface area and diverse porous structures, agrobasalt has a high capacity to retain nutrients and water [44,45,46], which can contribute to improving soil properties. Chemical modification of basalt rocks can generate new minerals, such as clay minerals and zeolites, which can be effective in environmental remediation [47].

The characteristics of adsorbents described above make them valuable materials for improving the structural, physical, and chemical properties of soils, thereby enhancing their biological activity. The selection of an effective adsorbent and soil revitalization method is extremely important, as shaping the physical and chemical properties of soils can stimulate the activity of soil enzymes, whose main source is microorganisms. Enzyme activation can accelerate the conversion of petroleum-derived products, as they are effective catalysts for many reactions. So far, the response of soil enzyme pools, particularly those predominantly representing the oxidoreductase and hydrolase classes, has not been extensively analyzed. The innovative aspect of this study is also reflected in the comprehensive analysis of the relationships between soil enzyme activity and its physicochemical properties and the sensitivity of *Zea mays* in soil exposed to the pressure of the analyzed pollutants. Therefore, the aim of our research was to determine the potential of vermiculite and agrobasalt in mitigating the effects of soil contamination with petroleum-derived products. The research hypotheses focused on answering the following questions: (1) What is the effect of vermiculite and agrobasalt on soil enzymes and the yield of *Zea mays*? (2) Can these sorbents improve the productivity of soil destabilized by diesel oil and unleaded gasoline?

## 2. Results

### 2.1. The Role of Vermiculite and Agrobasalt in Maintaining the Enzymatic Activity of Gasoline-Contaminated Soil

**Figure 1:**
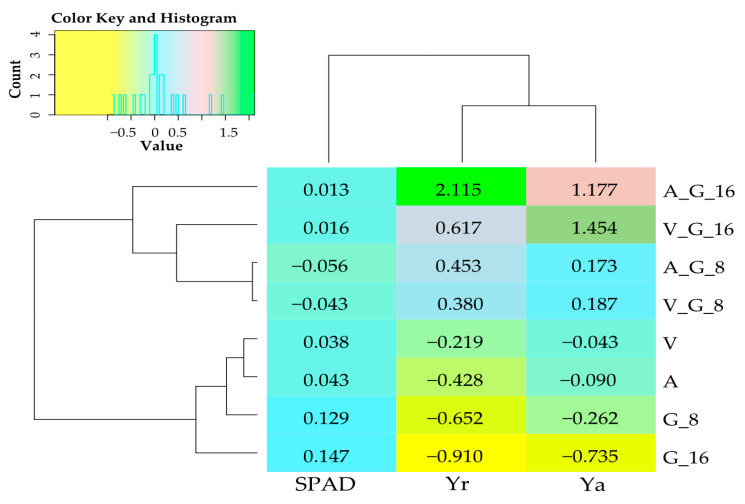
Index of the effect of unleaded gasoline (G), vermiculite (V) and agrobasalt (A) on *Zea mays* yield (Yr, Ya) and leaf greenness index (SPAD), 8 and 16 – doses of G (cm^3^ kg^−1^ d.m. of soil). Explanations of the abbreviations are provided in Table 1.

**Scheme 1:**
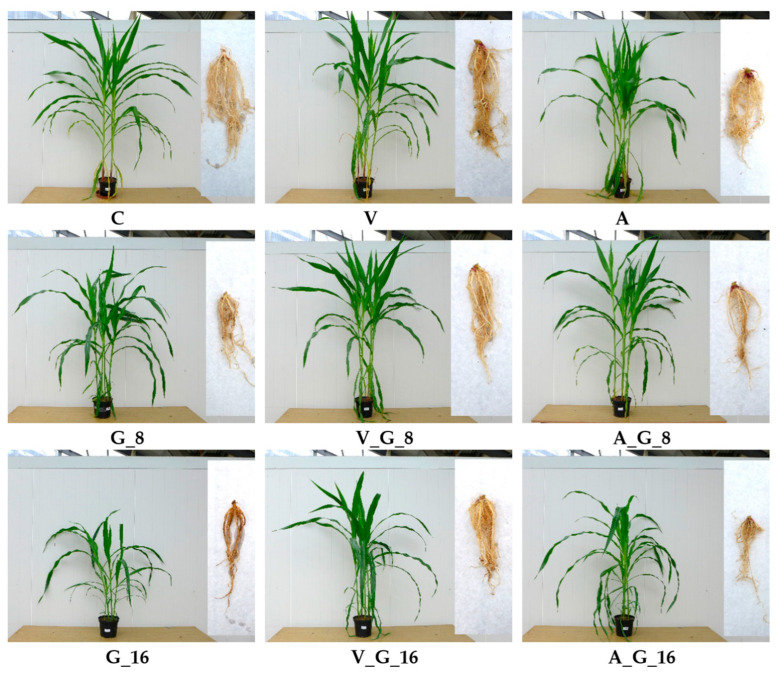
Plants tested on the day of harvest, gasoline (G), vermiculite (V), and agrobasalt (A), 8 and 16—doses of gasoline.

Contamination of the soil with 95 unleaded gasoline (G) significantly disrupted the growth and development of *Zea mays* (Table 1, Scheme 1). This product, applied to the soil at a rate of 16 cm^3^ kg^−1^ d.m. of soil, reduced the biomass of the aerial parts (Ya) by 73.5% and the roots (Yr) by 91%. The application of vermiculite (V) and agrobasalt (A) to the soil not exposed to G not only did not stimulate the growth and development of *Zea mays* but actually had a negative effect on the biomass of the aerial parts and roots, causing a reduction in Ya by 4% and Yr by 22% in the V series, and by 9% and 43% in the A series, respectively. Conversely, in soil contaminated with G, both vermiculite and agrobasalt induced the growth and development of the aerial parts and roots, as illustrated by the positive impact indices of both sorbents on Ya and Yr as shown in Figure 1. The potential of the sorbents was particularly evident in the samples exposed to 16 cm^3^ G kg^−1^ d.m. of soil, where the impact index (IF) of vermiculite on Ya was 1.45 and on Yr was 0.62, and for agrobasalt, it was 1.78 and 2.11, respectively. Generally, the effect of agrobasalt in soil under G pressure was stronger than that of vermiculite.

The impact of G on *Zea mays* resulted in changes in the leaf greenness index (SPAD) values (Table 1). Unleaded gasoline in unsupplemented soil significantly increased SPAD values. In contrast, vermiculite and agrobasalt, regardless of the G dose, had relatively little effect on SPAD values, as confirmed by IF values ranging from −0.056 (A_G_8) to 0.043 (A) (Figure 1).

**Figure 2:**
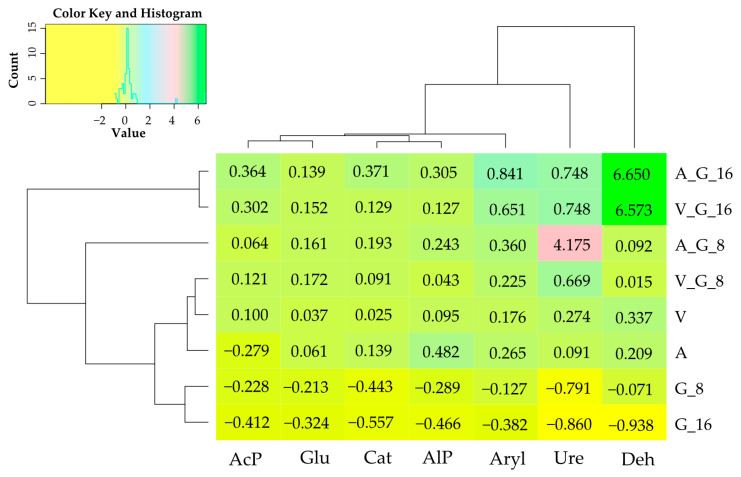
Index of the effect of unleaded gasoline (G), vermiculite (V), and agrobasalt (A) on soil enzyme activity, 8 and 16—doses of G (cm^3^ kg^−1^ d.m. of soil). Explanations of the abbreviations are provided in Table 2.

The analysis of seven soil enzymes yielded a comprehensive dataset characterizing soil conditions, thereby enhancing the innovative significance of the experiment. Gasoline had a negative effect on soil enzyme activity (Table 2). The higher the gasoline content in the soil, the greater the inhibition of enzyme activity. On average, regardless of the sorbent used, the inhibition of enzyme activity by the higher dose of gasoline was as follows: Deh by 71.8%, Cat by 51%, Ure by 81%, AcP by 24%, AlP by 49%, Glu by 28%, and Aryl by 19%. These figures show that gasoline destabilized the activity of Ure and Deh the most, and Aryl and AcP the least. The average results, independent of the gasoline dose, obtained in the series with vermiculite and agrobasalt indicate their positive effect on soil enzyme activity under conditions of gasoline contamination. The activity of all enzymes except AcP in the agrobasalt series was significantly higher than in the control without these sorbents. The effectiveness of vermiculite and agrobasalt in restoring the activity of enzymes destabilized by gasoline is demonstrated by the data presented in Figure 2. The effect indices of gasoline on the activity of the individual enzymes were always negative, whereas after the introduction of vermiculite and agrobasalt into the soil, they assumed positive values, although agrobasalt reduced the AcP index to −0.279 in the uncontaminated soil. The most remarkable effects of vermiculite and agrobasalt were observed on Deh in soil with 16 cm^3^ gasoline kg^−1^ and of agrobasalt alone on Ure in soil with 8 cm^3^ gasoline kg^−1^.

The implementation of soil G caused relatively little change in the physicochemical properties, as evidenced by the average results, regardless of sorbent supplementation (Table 3). It had no effect on EBC, CEC, Eh, and specific density and caused a reduction in C_org_, N_Total_, and C:N ratio and an increase in pH and EC value. The application of vermiculite and agrobasalt induced larger changes in soil properties. Analyzing the average results, irrespective of the degree of soil G contamination, it was found that the sorbents contributed to a significant increase in C_org_ content, pH value, EBC, CEC, BS, EC, and C:N ratio. The sorbents had opposing effects on the soil N_Total_ conten;, i.e., agrobasalt increased the amount of N_Total_ in relation to the sorbent soil, whereas vermiculite decreased it.

### 2.2. The Role of Vermiculite and Agrobasalt in Maintaining the Enzymatic Activity of Diesel Oil-Contaminated Soil

**Scheme 2:**
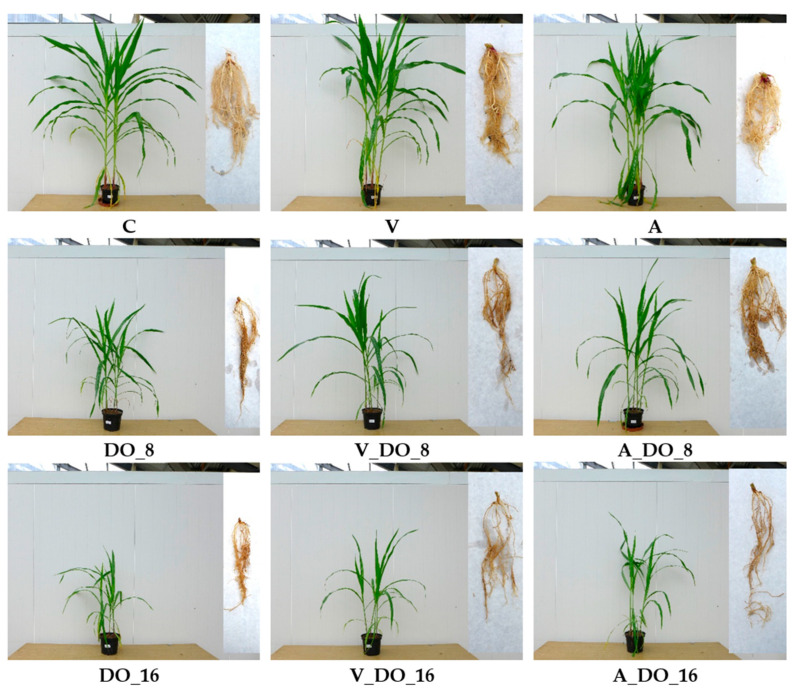
Plants tested on the day of harvest, diesel oil (DO), vermiculite (V), and agrobasalt, (A), 8 and 16—doses of diesel oil.

Contamination of soil with diesel oil (DO) significantly disrupted the growth and development of *Zea mays*, as reflected in a significant reduction in aerial biomass (Ya) and roots (Yr) (Table 4, Scheme 2).

**Figure 3:**
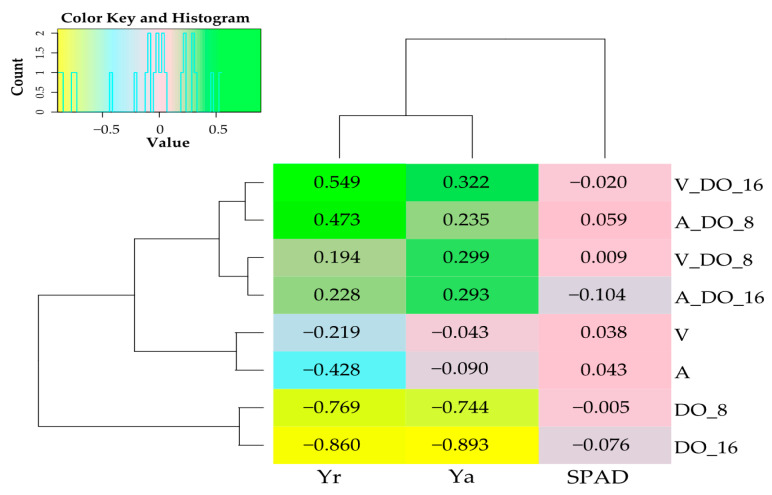
Index of the effect of diesel (DO), vermiculite (V), and agrobasalt (A) on *Zea mays* yield (Yr, Ya) and leaf greenness index (SPAD), 8 and 16—doses of DO (cm^3^ kg^−1^ d.m. of soil). Explanations of the abbreviations are provided in Table 4.

The negative effect of DO on *Zea mays* biomass was observed in all experimental series, regardless of sorbent supplementation. This effect was more pronounced with higher levels of soil contamination with DO. The greatest reduction in biomass Ya (by 89%) and Yr (by 86%) was observed in soil under the pressure of 16 cm^3^ DO kg^−1^ d.m. of soil. Positive, though not always statistically significant, effects of sorbents on *Zea mays* biomass were observed in soil contaminated with DO. This is confirmed by the influence indices of vermiculite and agrobasalt on Ya and Yr as shown in Figure 3, which ranged from 0.235 (A_DO_8) to 0.322 (V_DO_16) for Ya in soil under DO pressure and from 0.194 (V_DO_8) to 0.473 (A_DO_8) for Yr. The influence of the studied variables on the SPAD index was relatively small, as only the dose of 16 cm^3^ DO kg^−1^ d.m. of soil had a destructive effect on this index, regardless of the application of vermiculite and agrobasalt (Table 4, Figure 3).

**Figure 4:**
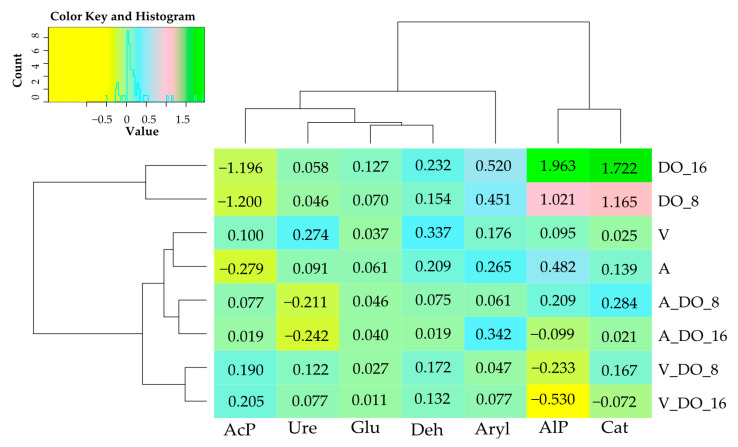
Index of the effects of diesel oil (DO), vermiculite (V), and agrobasalt (A) on soil enzyme activity, 8 and 16—doses of DO (cm^3^ kg^−1^ d.m. of soil). Explanations of abbreviations are provided in Table 5.

The response of soil enzymes to soil contamination with DO and the sorbents used was inconclusive (Table 5). DO stimulated the activity of the following enzymes with applied sorbents and in soil without sorbents Deh, Cat, AlP, Glu, and Aryl, while inhibiting the activity of AcP and Ure, although in the case of the latter enzyme only in the series with vermiculite and agrobasalt. In soil without sorbents, the effect index of DO on all enzymes except AcP was positive and increased with higher doses as shown in Figure 4. The IF values in the soil of the V_DO_8 object indicate that vermiculite in soil with 8 cm^3^ DO kg^−1^ increased the activity of all enzymes, except for AlP, for which the IF of vermiculite was negative at −0.233. This negative effect was even more pronounced in soil with 16 cm^3^ DO (IF = −0.530). In the series with the higher DO dose, the negative IF of vermiculite was also observed for Cat activity (−0.072). In contrast, the IF of agrobasalt on Ure activity was negative in soil with both DO doses and positive for the other enzymes, but only in soil with 8 cm^3^ DO, as in soil with 16 cm^3^ DO, a negative IF of agrobasalt also occurred for AlP.

Soil contamination with DO contributed to changes in certain physicochemical properties (Table 6). The average results, regardless of vermiculite and agrobasalt application, indicate that these changes were associated with the contamination level, with higher DO content in the soil causing greater fluctuations. Under the influence of 16 cm^3^ DO kg^−1^ d.m. of soil, the content of C_org_ increased by 3.0 g kg^−1^ d.m. of soil, N_Total_ by 0.12 g kg^−1^ d.m. of soil, EBC by 11.4 mmol(+) kg^−1^ d.m. of soil, CEC by 4.2 mmol(+) kg^−1^ d.m. of soil, EC by 79.5 µS cm^−1^, and BS by 9.9%. The C:N ratio also increased by 1.5, and the pH value increased by 0.6, thereby reducing HAC by 7.3 mmol(+) kg^−1^ d.m. of soil. A reduction in Eh of 37.4 mV was also noted. Physicochemical properties also changed under the influence of vermiculite and agrobasalt. On average, regardless of the degree of soil contamination with DO, vermiculite caused an increase in C_org_ content by 0.4 g kg^−1^ d.m. of soil, pH by 0.1, HAC by 2.0 mmol(+) kg^−1^ d.m. of soil, EBC by 2.7 mmol(+) kg^−1^ d.m. of soil, CEC by 4.7 mmol(+) kg^−1^ d.m. of soil, and EC by 18.5 μS cm^−1^, while agrobasalt increased C_org_ content by 0.5 g kg^−1^ d.m. of soil, pH by 0.1, HAC by 0.7 mmol(+) kg^−1^ d.m. of soil, EBC by 12.3 mmol(+) kg^−1^ d.m. of soil, CEC by 13.0 mmol(+) kg^−1^ d.m. of soil, EC by 11.2 μS cm^−1^, and BS by 4.4%. Both vermiculite and agrobasalt reduced N_Total_ content but increased the C:N ratio.

### 2.3. Interactions Between Biochemical and Physicochemical Properties of Soil Exposed to DO and G with Vermiculite and Agrobasalt

**Figure 5:**
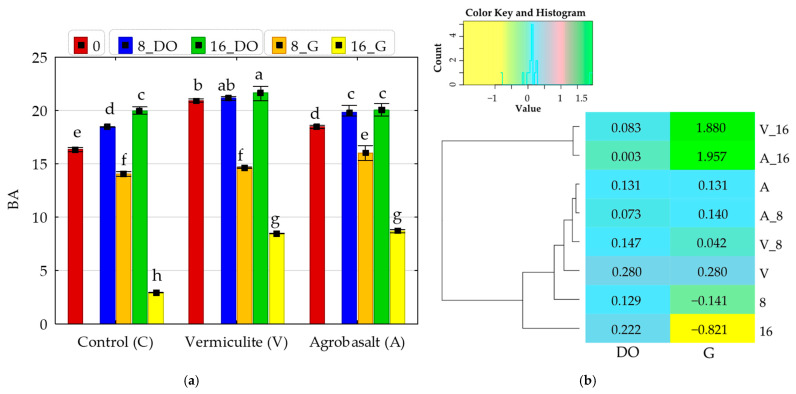
Soil biochemical quality—BA (**a**) and index of effect of diesel (DO), unleaded gasoline (G), vermiculite (V), and agrobasalt (A) on BA (**b**), 8 and 16—doses of G and DO (cm^3^ kg^−1^ d.m. of soil). The same letters (a–h) within each property indicate a homogeneous group, *p* < 0.050, N for each standard deviation = 4.

The biochemical soil quality index (BA) effectively reflects the contrasting effects of petroleum-derived products on soil enzyme activity (Figure 5a,b). Diesel oil (DO) increased the BA index in both control and sorbent-amended soils, whereas gasoline (G) decreased it. Specifically, DO at 8 cm^3^ kg^−1^ d.m. of soil without sorbent amendments increased the BA value by 13%, and at 16 cm^3^ DO kg^−1^ d.m. of soil, the increase was 22%. Conversely, G reduced BA by 14% and 82%, respectively. Supplementation of uncontaminated soil with vermiculite increased BA by 28% and agrobasalt by 13%. Both vermiculite and agrobasalt induced more significant changes in samples contaminated with G than with DO. A significant increase in BA was observed in soil exposed to 16 cm^3^ G kg^−1^ dry soil, where the influence index of vermiculite was 1.880, and that of agrobasalt was 1.957.

**Figure 6:**
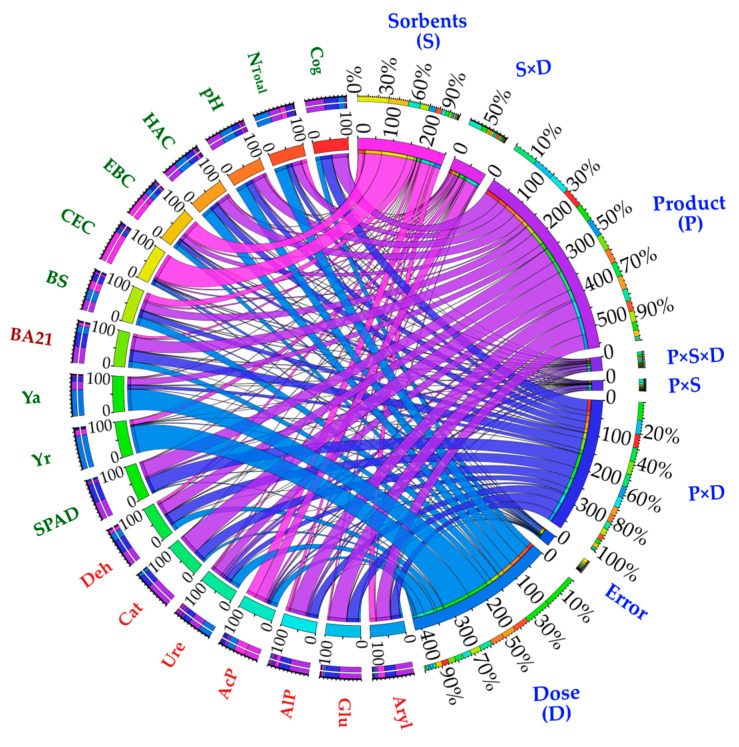
The loadings of all independent variables (type of petroleum product, its dosage, and type of sorbent) in explaining the dependent variables (soil physicochemical properties, SPAD, biomass of aerial parts and roots of *Zea mays*, activities of seven soil enzymes) are given in percentages. The explanations of the abbreviations are given in Table 1, Table 2 and Table 3.

The interactions between the activity of the seven enzymes, the physicochemical properties of soil contaminated with the analyzed xenobiotics, and the sensitivity of *Zea mays* to their pressure have not been sufficiently explored to date. Of all the independent variables, the biomass of aerial parts (63%) and roots (80%) of *Zea mays* and Ure activity (30%) were most influenced by the dose of petroleum products, while SPAD (43%), Deh (35%), Cat (60%), AlP (48%), Aryl (43%), Glu (53%), BA (39%), C_org_ (46%), N_Total_ (27%), pH (36%), HAC (33%), and BS (26%) by the type of petroleum product, and on AcP (29%), EBC (48%) and CEC (75%)—sorbents as shown in Figure 6.

**Figure 7:**
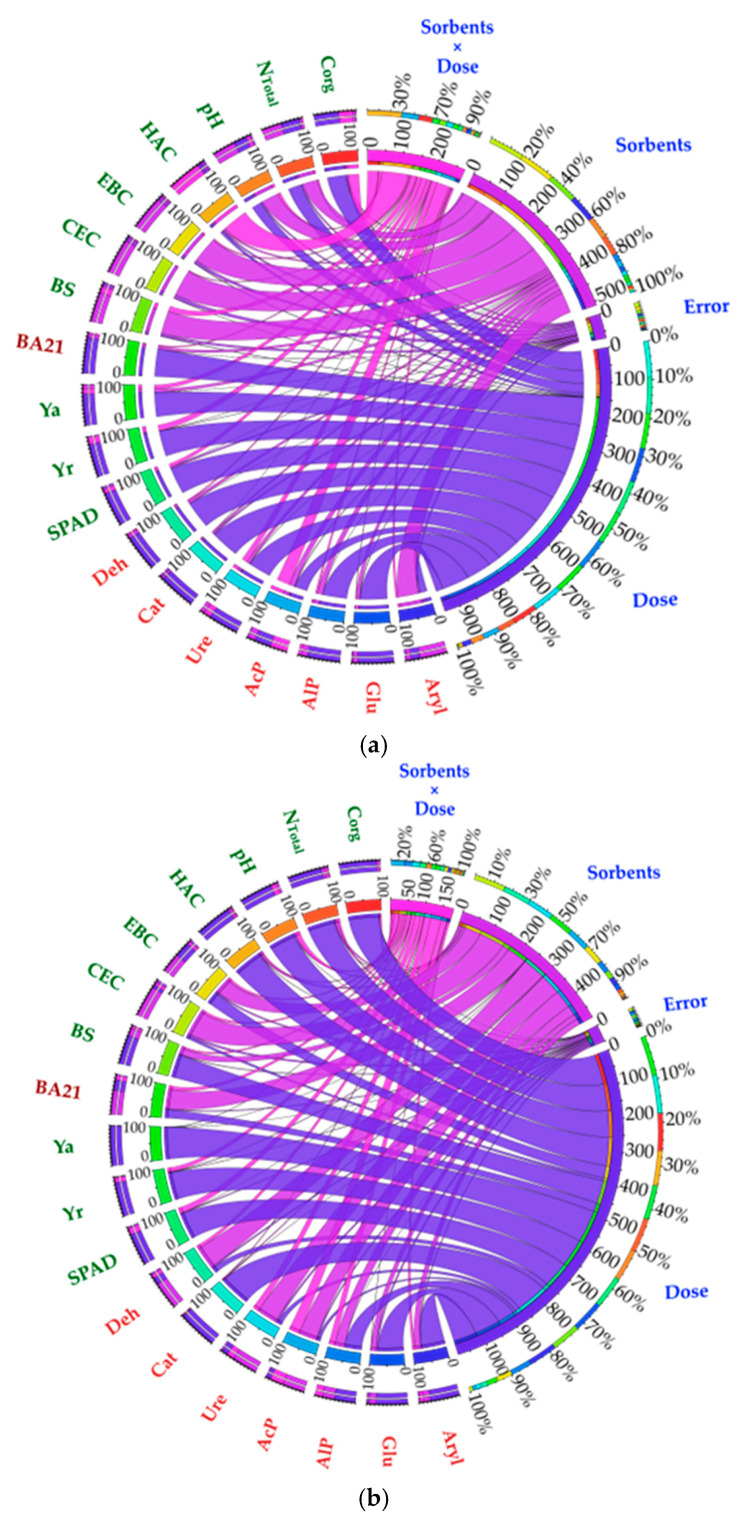
The loadings of the independent variables (type of sorbent and dosage of petroleum product) in explaining the dependent variables (soil physicochemical properties, SPAD, biomass of aerial parts and roots of *Zea mays*, activities of seven soil enzymes), in percentages, broken down by the series with unleaded gasoline (**a**) and diesel oil (**b**). The explanations of the abbreviations are given in Table 1, Table 2 and Table 3.

Given that most dependent variables were predominantly influenced by the type of petroleum product, it was decided to test the percentage contribution of the independent variables (η^2^) in shaping the studied parameters separately in the series contaminated with gasoline (G) (Figure 7a) and diesel oil (DO) (Figure 7b). In both series exposed to DO and G, the growth and development of *Zea mays*, measured by Ya, Yr, and SPAD, were mainly determined by the dosage of these petroleum products. This variable accounted for 80–99% of plant growth and development in soil contaminated with DO and 77–83% in soil contaminated with G. Regarding soil properties, the dosage of DO and G had a greater influence on Cat, AlP, Glu, Corg, and HAC, while the sorbent had a more significant effect on EBC and CEC. Different effects of these independent variables were observed for Deh, Ure, AcP, Aryl, N_Total_, pH, and BS. Specifically, the gasoline dosage had a more pronounced impact on Deh, Ure, and AcP activities than the DO dosage. Conversely, sorbents had a stronger influence on Aryl activity, N_Total_ content, pH level, and BS value in soil contaminated with DO, compared to soil treated with G.

**Figure 8:**
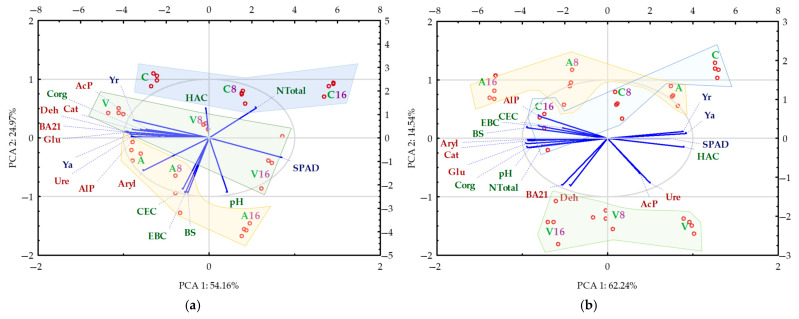
Relationship between the effects of unleaded gasoline (**a**) and diesel oil (**b**) and the properties studied (soil physicochemical properties, SPAD, biomass of aerial parts and roots of *Zea mays*, activities of seven soil enzymes), presented using PCA. The explanations of abbreviations are given in Table 1, Table 2 and Table 3.

There was a strong positive correlation between *Zea mays* biomass, the activity of all enzymes, BA index, and Corg content, and between CEC, EBC, BS, and Aryl in the soil where gasoline exposure was tested (Figure 8a, Table S1). There was a significant negative correlation between enzyme activity, soil organic carbon content, and maize yield and the magnitude of the SPAD index. Soil pH and HAC and N_Total_ and BA, CEC, EBC, and BS were also negatively correlated. The vectors representing the primary variables in Figure 8b, which illustrates the results of the diesel oil experiment series, were arranged slightly differently. Specifically, the activities of Ure and AcP were positively correlated with each other and negatively correlated with Cat, Glu, Aryl, and AlP, as well as with CEC, EBC, and BS. The biomass of *Zea mays* was negatively correlated with the content of C_org_ and N_Total_ and with the activities of Deh, Cat, AlP, Glu, and Aryl, as well as with the values of EBC, BS, BA, and pH. The SPAD index of maize leaves was positively correlated with its biomass in this series of experiment.

**Figure 9:**
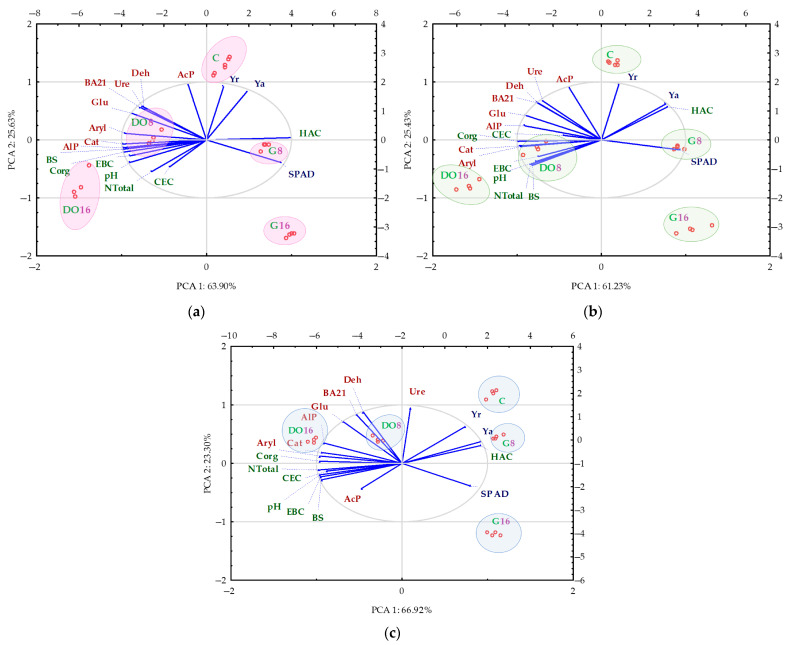
Relationships between the effects of unleaded gasoline (G) and diesel oil (DO) on the properties studied (soil physicochemical properties, SPAD, biomass of aerial parts and roots of *Zea mays*, activities of seven soil enzymes) in the control series (**a**), in the series with vermiculite (**b**), and in the series with agrobasalt (**c**), presented using PCA. The explanations of the abbreviations are given in Table 1, Table 2 and Table 3.

Comparison of the distribution of vectors in the individual quadrants of the coordinate system indicates that both vermiculite and agrobasalt, although influencing the parameters studied, did not fully restore the stability of soil disrupted by petroleum products (Figure 9a–c). Some vectors representing the primary dependent variables were positioned in the same quadrants regardless of the sorbent used. In all figures, the vectors for Yr, Ya, and HAC were located in the first quadrant, the vectors for BA, Deh, and Glu in the second quadrant, BS, EBC, N_Total_, and pH in the third quadrant, and SPAD in the fourth quadrant. Shifts in the positioning of vectors in the coordinate system pertain to Cat, AlP, AcP, Ure, Aryl, and C_org_.

**Figure 10:**
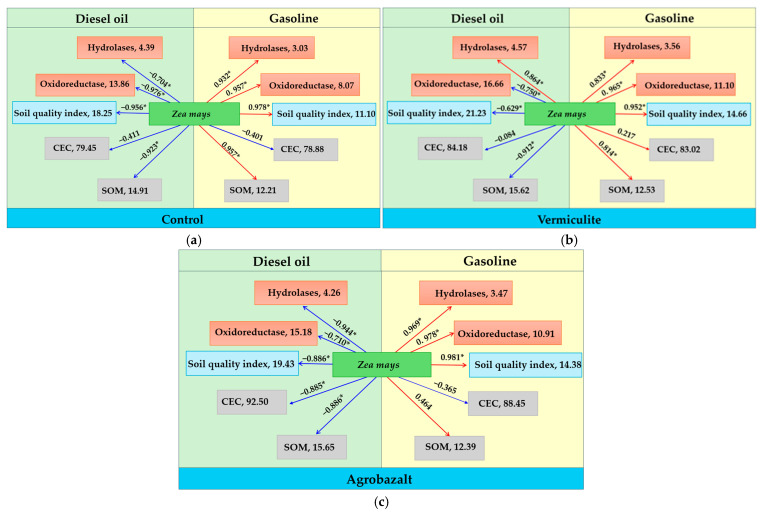
Relationships between the biomass of *Zea mays* shoots and the activity of oxidoreductases and hydrolases, the soil quality index, soil organic matter (SOM), and soil cation exchange capacity (CEC) in control soil—unsupplemented with sorbents (**a**), with vermiculite (**b**), and with agrobasalt (**c**), determined by correlation coefficients. Red arrows indicate positive correlations, while blue arrows indicate negative correlations. The numbers next to the arrows represent the effect size of the relationships. *—*p* < 0.05, N = 12. The numbers next to the names of the parameters tested are the means for the traits studied.

Summarizing the results obtained, it can be stated that diesel oil (DO) caused a negative correlation between the activities of hydrolases, oxidoreductases, the soil quality index, CEC, and SOM with the yield of *Zea mays* as shown in Figure 10. In contrast, gasoline (G) induced a positive correlation between the biomass of *Zea mays* and these parameters. An exception is a correlation between CEC and maize yield in the control series and with agrobasalt.

Concluding the discussion on the destabilization of soil homeostasis by petroleum products, it can be stated that soil contamination with gasoline caused greater perturbations than with diesel oil (Figure 10a). This is evidenced by the lower activity of oxidoreductases and hydrolases, lower soil quality index, and lower SOM content in soil contaminated with G compared to DO. Both vermiculite (Figure 10b) and agrobasalt as shown in Figure 10c mitigated the negative impact of petroleum products, but better effects were achieved by applying vermiculite to the soil. In soil degraded by G, vermiculite increased the soil quality index from 11.10 to 14.66, and agrobasalt from 11.10 to 14.38, while in soil degraded by DO, the increases were from 18.25 to 21.23 and from 18.25 to 19.43, respectively.

## 3. Discussion

### 3.1. Degradation of the Soil Environment by Diesel Oil and Unleaded Gasoline

Contamination of the environment with petroleum products is a global problem [3,19,48]. The fact that these products can persist in the soil and significantly disrupt its functions [49], thereby degrading the quality of agricultural products and water, which adversely affects human health, remains a pressing concern that continues to capture societal interest [50,51]. The release of hydrocarbon substances into the environment leads to partial soil degradation, which in extreme cases can lead to the removal of soils from agricultural production [52]. In our studies, the induced changes in physicochemical properties were dependent on the type of petroleum product and the level of contamination. Generally, greater perturbations in physicochemical properties (EBC, CEC, and BS) were caused by diesel oil (DO) than by gasoline (G). This stronger effect of DO on soil physicochemical properties may be due to the adsorption of a greater amount of DO on the soil mineral surfaces, compared to G [53,54]. Moreover, soil contaminated with G, as opposed to DO, contributed to a decrease in C_org_, N_Total_, and the C:N ratio. This differential effect of the tested pollutants was probably due to their distinct structures and properties [55,56] and consequently their different lability and susceptibility to microbial degradation [57,58]. Some of these pollutants may even undergo permanent immobilization [9], thereby disrupting abiotic and biotic interactions and altering the physical, chemical, and biological properties of the soil [59,60].

Deterioration of water-air conditions and reduced permeability [50,59] inhibit plant growth and development [61]. In our studies, soil contamination with both petroleum products disrupted the growth of *Zea mays*, resulting in a significant reduction in plant biomass. The hydrocarbons in diesel oil (DO) had a more toxic effect on maize, compared to the hydrocarbons in gasoline (G). Diesel oil consists mainly of paraffinic, naphthenic, and aromatic hydrocarbons, whereas unleaded gasoline is composed of aliphatic hydrocarbons [62]. According to Grifoni et al. [63], the molecular weight and hydrophobicity of these products determine their solubility in water, which directly affects their uptake by plants. The stronger negative effect of DO compared to G on *Zea mays* observed in our studies could be attributed to the lower solubility of higher molecular weight (HMW) hydrocarbons in water, compared to lower molecular weight (LMW) hydrocarbons [64]. In soil degraded by petroleum products, water and nutrient uptake is impaired [65]. Therefore, it is crucial to select fast-growing plants with well-developed root systems to restore such soils [5,66,67,68]. Additionally, these plants should produce a large amount of biomass [54,69], which can then be utilized for energy purposes [26,70,71]. Such plants include *Zea mays*, which has been used in our studies for phytoremediation of soil degraded by DO and G, as well as *Lolium perenne* L. × *Hybridum, Poa pratensis*, *Festuca rubra*, *F. arundinacea, Phleum pratense*, *Dactylis glomerata* [5,72,73,74], and *Avena sativa*, which has been used in previous studies by the authors.

Soil contamination by petroleum products leads to the deterioration of soil functions, resulting in disturbances in soil enzyme activities [75,76,77], which are valuable bioindicators used in ecological risk assessment [10,73,78,79]. The extent to which soil enzyme properties are disturbed depends on the degree of pollution, type of pollutant, duration of pollutant presence, soil grain size, and nutrient content [28,75,80]. In our studies, the activity of five out of the seven enzymes (Deh, Cat, AlP, Aryl, Glu) was predominantly determined by the type of petroleum product, with only the activity of Ure being more strongly influenced by the level of soil contamination (Table 2 and Table 5). Diesel oil (DO) stimulated the activity of all enzymes except AcP, whereas gasoline (G) inhibited the activity of all enzymes. These changes were well reflected in the biochemical soil quality index (BA), which includes enzymes from the oxidoreductase and hydrolase classes. The lowest IF value associated with G corresponds to the highest dose of this contaminant, with an IF = −0.821, while for DO, the IF = 0.222 as shown in Figure 2 and Figure 3. Similarly, the obtained IF values for the contaminants revealed a particular sensitivity of Deh to G pressure (IF = −0.938; G_16), while for DO, the highest stimulation was observed for AlP (IF = 1.963; DO_16) and Cat (IF = 1.722; DO_16). Oxidoreductases initiate the conversion of petroleum products [81]. The activity of dehydrogenases and catalase is considered by some authors [10,77,82,83] to be a marker of oil degradation dynamics.

In our studies, the application of DO to the soil increased the value of the BA index, while G decreased it. The differences in the effects of DO and G on soil enzymes stem from variations in the strength of direct toxic interactions of the hydrocarbons present in petroleum products [10]. Hydrocarbons in gasoline degrade microbial cytoplasmic membranes more rapidly than those in diesel oil [83,84,85], thus contributing more intensely to the destabilization of microorganisms, which are the main source of soil enzymes [75,76].

### 3.2. Effectiveness of Vermiculite and Agrobasalt in Revitalizing Soils Contaminated with Diesel and Unleaded Gasoline

The consequences of soil contamination with petroleum-derived substances, including gasoline and diesel oil, are undoubtedly related to a reduction in soil permeability, maximum density, and optimum moisture content [86]. In our studies, these xenobiotics were also found to cause significant changes in the physicochemical properties of the soil. The scale of changes was determined by the type of petroleum product, which was not observed in relation to the soil amendments implemented. Both vermiculite and agrobasalt induced an increase in all parameters (C_org_ content, pH, EBC, CEC, BS, and C:N ratio), except for the N_Total_ content (Table 3 and Table 6). An increase in Corg content and the C:N ratio could be expected due to the polymerization process of organic carbon on mineral surfaces, which is a necessary step for its accumulation in the soil, ensuring stability and bioavailability [87,88]. Protection against degradation is provided by sorption, co-precipitation, and complexation processes, which ultimately lead to mineral–organic carbon association [88,89]. The decrease in N_Total_ content by sorbents was probably a result of high mineralization of organic nitrogen [90].

When assessing the toxicity scale of petroleum-derived substances, the complexity of their chemical structure must be considered, as it determines processes such as adsorption, phototransformation, and biological transformation in soils [57]. Ultimately, their interference with soil condition also translates into destabilization of the growth and development of plants cultivated in it [61]. One of the symptoms of the adverse effects of gasoline (G) and diesel oil (DO) on soil quality, observed in the studies, was a significant disturbance in both the growth and development of *Zea mays*. Therefore, it was justified to undertake soil revitalization techniques by using adsorbent materials such as vermiculite and agrobasalt [91]. In soil subjected to a pressure of 16 cm^3^ of G or DO kg^−1^ d.m. of soil, both vermiculite and agrobasalt induced growth and development of aerial and root parts of plants (Table 1 and Table 4, Figure 1 and Figure 3, Photography 1 and 2). However, the effect of agrobasalt in soil was more favorable than that of vermiculite, irrespective of the type of contamination applied. This is reflected in the IF values for agrobasalt concerning the analyzed parameter. In soil contaminated with G, the values were 2.115 for Yr and 1.177 for Ya. Conversely, in the series without the sorbent, negative IF values were observed, specifically −0.910 for Yr and −0.735 for Ya. The positive response of plants to the applied adsorbent probably corresponded to an increase in the soil content of P, K, C, and Mg, sourced from basalt [92]. For this reason, in the studies by Conceicao et al. [93] a 200% increase in the aerial biomass of *Zea mays* was achieved after applying basalt dust. Furthermore, this sorbent induced an increase in soil pH value, similar to vermiculite. This tendency is favored by the reaction of Ca and Mg carbonates with hydrogen in soil amended with basalt. The reaction of carbonic acids with silicate minerals leads to the release of alkaline cations Ca^2+^ and Mg^2+^, resulting mainly in the synthesis of bicarbonate ions (HCO_3_^−^) and, to a lesser extent, carbonate ions (CO_3_^2−^) [94,95]. It is also worth emphasizing that raising soil pH neutralizes the toxicity of Al, which significantly influences global agricultural production by compartmentalizing in plant root systems, excessively inducing reactive oxygen species (ROS) formation, and higher lipid peroxidation [96]. Considering that agrobasalt is a natural, clayey silicate material [40], and vermiculite is a silicate mineral, whose basic planes consist of six-membered SiO_4_ rings [97], it might be expected that these sorbents would stimulate the growth and development of *Zea mays*. Si induces plant resistance to xenobiotics by depositing as solid amorphous silica in plant cell walls, enhancing their structural integrity and promoting the synthesis of organic defense compounds [91,98]. However, in order to fulfill its expected function, silicic acid must undergo a slow polymerization process in the plant root, followed by translocation to the aerial part of the plant with the involvement of the transporters Lsi1 and Lsi2 [99].

The scale of soil disturbances under the pressure of the analyzed petroleum-derived substances is also precisely defined based on the response of biochemical parameters, which react rapidly to alarming changes in this ecosystem [92]. Although soil contamination with gasoline (G) inhibited the activity of all soil enzymes analyzed, the greatest sensitivity to this xenobiotic can be attributed to Ure and Deh. This sensitivity was expressed at levels of 81.3% and 71.8%, respectively. Unprecedentedly, it is precisely towards these two enzymes that the highest bioremediation potential of vermiculite and agrobasalt was revealed. The IF values obtained for both sorbents also highlight this trend, with the highest values recorded for Deh (IF = 6.650; A_G_16 and IF = 6.573; V_G_16) in soil subjected to a 16 mg G kg^−1^ d.m. of soil. Ultimately, this resulted in the highest IF values for vermiculite and agrobasalt, assigned to the BA values in soil treated with the highest dose of G, which were 1.880 and 1.957, respectively. This trend probably corresponds to the increase in soil pH values in the presence of both sorbents. For vermiculite, this is generated by the presence of water and hydrated Mg^2+^ in the interlayer spaces as well as hydroxide anion (OH^–^) in the octahedral sheets [97]. In the case of Deh, a conformational change occurs that facilitates enzymatic catalysis by destabilizing hydrogen bonds in the enzyme’s center [100]. Kappaun et al. [101] state that Ure activity increases significantly at pH 7–8. Since Deh is a constitutive element of the enzymatic system of living microorganisms, closely dependent on their activity [102], the trends obtained were likely generated by the ability of both sorbents to retain nutrients in the soil [103]. The fact that both vermiculite and agrobasalt were able to mitigate the toxic effects of G on other soil enzymes was dictated by their ability to condition mineral surfaces, thanks to exchangeable cations in their interlayers. This results in a greater number of absorption sites for cation–π bonds with petroleum compounds [104]. Therefore, not without significance for the effectiveness of vermiculite is its structure, consisting of two tetrahedral sheets composed of Si^4+^ and Al^3+^ cations, between which an octahedral sheet is placed [97]. Substitutions of cations in the tetrahedral position generate a much larger number of Brønsted acidic sites on the surface than in other mineral sorbents [105]. Potential sites for ion exchange are crystalline defects located at the edges or basal planes, including six-membered rings of SiO_4_ or AlO_4_ in vermiculite [97]. However, in the series with DO, neither vermiculite nor agrobasalt fulfilled the expected bioremediation function towards AlP, unlike other enzymes, except for Cat (V) and Ure (A), as evidenced by the research results obtained by Borowik et al. [26]. As Khadem and Raiesi [106] argue, this could be due to the collocation between the substrate and the enzyme causing inhibition of AlP activity, due to changes in the structure of the functional groups.

## 4. Materials and Methods

### 4.1. Research Project

**Figure 11:**
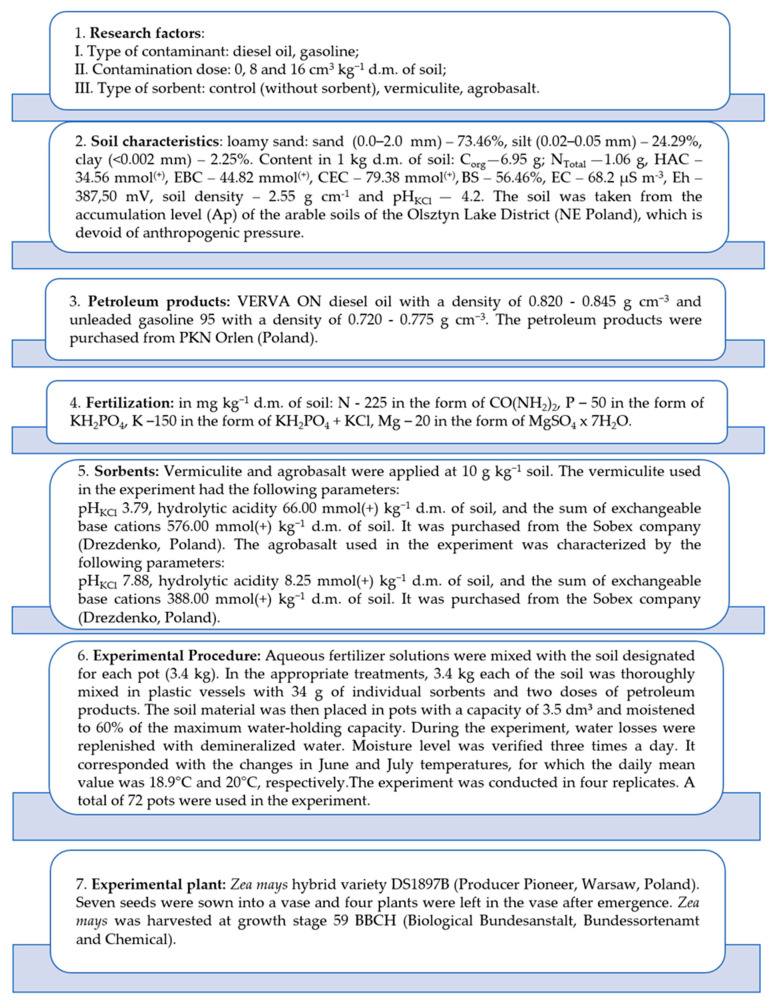
Highlights of the research.

A vegetation experiment was conducted in the north-eastern part of Poland (53.72° N, 20.42° E) in a vegetation hall, using a split-plot design. The pot experiment was carried out in two series: the first series consisted of soil contaminated with unleaded gasoline (36 pots), and the second series consisted of soil contaminated with diesel oil (36 pots). A total of 72 pots were used in the experiment, each containing 3.4 kg of soil. Detailed characteristics of the petroleum products are available on the PKN Orlen website [107,108]. Two sorbents were used to restore the equilibrium of the contaminated soil: vermiculite and agrobasalt. The experimental design is presented in Figure 11.

On the 60th day of the experiment, the Spectrum Technologies, Inc. chlorophyll meter (KONICA MINOLTA, Inc., Chiyoda, Japan) was used to determine the Spectrum Green Index (SPAD) of *Zea mays*. Immediately after the measurement of SPAD, the above-ground parts of the maize were cut, and the roots were extracted from the soil. The biomass of *Zea mays* was dried at 60 °C in a Binder D-78532 oven (Binder GmbH, Tuttlingen, Germany). On the day of plant harvest, soil samples were also collected for enzymatic, physicochemical, and chemical analyses.

### 4.2. Methodology for Determining Soil Properties

In moist soil samples, both uncontaminated and contaminated with petroleum products, with and without the application of sorbents, the activity of seven enzymes related to the cycles of carbon, nitrogen, phosphorus, and sulfur was determined in four replicates (Table 7).

Dehydrogenase (Deh) activity was determined according to the method described by Öhlinger [109], catalase (Cat) activity according to Johnson [110], and the activities of *β*-glucosidase (Glu), acid phosphatase (AcP), alkaline phosphatase (AlP), urease (Ure), and arylsulfatase (Aryl) according to Alef and Nannipieri [111]. The absorbance of the reaction products of all enzymes, except Cat, was measured using a PerkinElmer Lambda 25 spectrophotometer (Waltham, MA, USA) at the following wavelengths: 400 nm (Glu), 410 nm (AcP, AlP, Ure), 420 nm (Aryl), and 485 nm (Deh). Catalase activity was determined by titration with potassium permanganate. Detailed assay procedures, including buffers, temperature, and incubation times, as well as enzyme reaction termination methods, have been presented in our previous research [112,113]. All biochemical analyses, except for catalase, were based on validated standard curves developed for the substrates: formazan (TFF) for Deh, ammonium sulphate, ((NH_4_)_2_SO_4_) for Ure, and p-nitrophenol (C_6_H_5_NO_3_) for AcP, AlP, Aryl, and Glu

Physicochemical and chemical analyses, performed in four replicates, were conducted on air-dried soil sieved through a 2 mm mesh. The particle size distribution of the soil was determined before the experiment by laser diffraction using a Malvern Mastersizer 3000 Laser Diffraction (Malvern, Worcestershire, UK). Both in the initial soil (before the experiment) and after its conclusion, the content of organic carbon (C_org_) and total nitrogen (N_Total_) was measured using a Vario MaxCube CN macroanalyzer (Hanau, Germany). HI 2300—EC/TDS/NaCl Bench Meter (Smithfield, VA, USA) was used to determine specific electrical conductivity (EC). Additionally, the redox potential (Eh) of the soil and pH were determined on a pH–METR CP–505 (Elmetron, Zabrze, Poland). Soil pH was determined in 1 mol KCl dm^−3^, as well as hydrolytic acidity (HAC) and the sum of exchangeable basic cations (EBC). The cation exchange capacity (CEC) and base saturation (BS) of the soil were calculated from HAC and EBC. These determinations were carried out using standard methods described in our previous studies [70].

### 4.3. Statistical Analyses

Statistical analysis of the data (at a significance level of α = 0.05) was performed using Statistica 13.3 software [114]. Initially, the results of enzyme activities and soil chemical and physicochemical properties were checked for normality using the Shapiro–Wilk test. Subsequently, a post-hoc analysis—Tukey’s HSD test—was used to determine the significance of statistical differences between the petroleum product dose and the soil properties studied. The relationships between the variables were determined separately for the series contaminated with diesel fuel and unleaded petrol on the basis of the Pearson correlation matrix.

In order to better assess the impact of diesel fuel and unleaded gasoline on soil properties in relation to vermiculite and agrobasalt application, impact indices (IF) of petroleum products and sorbents on plant growth, development, and soil enzyme activities were presented. The procedure for determining these indices and the formulae are described in detail in our previous research [28]. These indices were visualized on heat maps created using R v1.2.5033 software (Boston, MA, USA) [115] with R v3.6.2 addition [116] and a gplots library [117]. Additionally, the soil quality index (BA), based on the activities of enzymes involved in the cycles of C, N, P, and S discussed in the manuscript, was helpful in determining soil fertility. The methodology and formula for the BA index are detailed in our previous studies [118,119]. The percentage contribution of the independent variables (η^2^) was also determined and visualized using Circos [120]. All biochemical, chemical, and physicochemical properties were assessed using PCA in Statistica 13.3 software [114]. This approach enabled the analysis of results from various perspectives. All statistical data were analyzed according to QC/QA standards guidelines. Data were analyzed separately within each property indicate a homogeneous group, *p* < 0.050, N for each standard deviation = 4.

The effects of vermiculite and agrobasalt in soil contaminated with diesel and petrol were also visualized, presenting the relationships between the biomass of the aboveground parts of *Zea mays* and the activity of oxidoreductases and hydrolases, the soil quality index (BA) and soil organic matter (SOM) and soil sorption capacity (CEC).

## 5. Conclusions

In soil not exposed to the pressure of unleaded gasoline and diesel oil, vermiculite increased the activity of dehydrogenases by 34%, urease by 27%, arylsulfatase by 18%, and agrobasalt by 21%, 9%, and 27%, respectively. Additionally, agrobasalt increased the activity of alkaline phosphatase by 48% and decreased the activity of acid phosphatase by 28%, while vermiculite increased the activity of both phosphatases by 10%. Both sorbents increased the content of exchangeable cations and the sorption capacity in the soil. Both vermiculite and agrobasalt did not alter the biomass of aerial parts of *Zea mays* but decreased the biomass of roots. However, in soil under pressure from petroleum products, these sorbents significantly increased the biomass of both aerial parts and roots. Both pollutants destabilized the growth of maize, but diesel oil had a more toxic effect on this plant than unleaded gasoline. In turn, gasoline inhibited the activity of all tested soil enzymes, as evidenced by the negative indices (IF) of this product on enzymes and the low biochemical soil quality index (BA), while diesel oil stimulated the activity of all enzymes except AcP. Vermiculite and agrobasalt stimulated the enzymatic activity of soil contaminated with unleaded gasoline more than soil contaminated with diesel oil. The conducted studies demonstrate that the sorbents tested can accelerate the degradation of petroleum products through their beneficial effect on soil enzyme activity and by mitigating their toxic effect on plants. The effect of these sorbents is similar, and the choice for soil revitalization should be based on cost considerations. It would be interesting in the future to compare the combined effect of vermiculite and agrobasalt with other soil amendments.

## Figures and Tables

**Table 1:** *Zea mays* yield and leaf green index (SPAD) in soil contaminated with gasoline (G) (g d.m. of pot ^−1^).

Properties	Dose Gasoline (G), cm^3^ kg^−1^ d.m. of Soil	Sorbent (S)			Average
		Control (C)	Vermiculite (V)	Agrobasalt (A)	
Aerial parts (Ya), g d.m. of pot^−1^	0	74.6 ± 0.4 ^a^	71.4 ± 1.9 ^ab^	67.8 ± 1.7 ^bc^	71.3 ^I^
	8	55.0 ± 2.3 ^d^	65.3 ± 1.3 ^c^	64.6 ± 0.6 ^c^	61.7 ^II^
	16	19.7 ± 0.8 ^f^	48.5 ± 2.2 ^d^	43.0 ± 2.2 ^e^	37.1 ^III^
	Average	49.8 ^C^	61.7 ^A^	58.5 ^B^	
Root yield (Yr), g d.m. of pot^−1^	0	14.6 ± 0.8 ^a^	11.4 ± 0.9 ^b^	8.4 ± 0.8 ^c^	11.5 ^I^
	8	5.1 ± 0.6 ^d^	7.0 ± 0.3 ^c^	7.3 ± 0.4 ^c^	6.5 ^II^
	16	1.3 ± 0.1 ^e^	2.1 ± 0.2 ^e^	4.1 ± 0.4 ^d^	2.5 ^III^
	Average	7.0 ^A^	6.9 ^A^	6.6 ^A^	
Leaf green index (SPAD)	0	40.6 ±0.8 ^c^	42.2 ± 0.3 ^bc^	42.4 ± 0.5 ^bc^	41.7 ^III^
	8	45.9 ± 0.5 ^a^	43.9 ± 1.0 ^b^	43.3± 0.5 ^b^	44.4 ^II^
	16	46.6 ± 0.6 ^a^	47.3 ± 0.7 ^a^	47.2 ± 0.6 ^a^	47.0 ^I^
	Average	44.4 ^A^	44.5 ^A^	44.3 ^A^	

The same letters (a–f) within each property indicate a homogeneous group, A–C for the sorbent, I–III for the dose of the petroleum product, *p* < 0.050, N for each standard deviation = 4, and N for each property tested = 36.

**Table 2:** Enzyme activity in 1 kg of soil contaminated with unleaded gasoline (G) at 1 h.

Enzyme	Dose Gasoline (G), cm^3^ kg^−1^ d.m. of Soil	Sorbent (S)			Average
		Control (C)	Vermiculite (V)	Agrobasalt (A)	
Dehydrogenases (Deh), mM TFF	0	12.00 ± 0.26 ^c^	16.04 ± 0.11 ^a^	14.51 ± 0.11 ^b^	14.19 ^I^
	8	11.15 ± 0.17 ^d^	11.32 ± 0.06 ^d^	12.18 ± 0.57 ^c^	11.55 ^II^
	16	0.74 ± 0.00 ^f^	5.60 ± 0.03 ^e^	5.66 ± 0.14 ^e^	4.00 ^III^
	Average	7.97 ^C^	10.99 ^A^	10.78 ^B^	
Catalase (Cat), M O_2_	0	0.16 ± 0.00 ^a^	0.16 ± 0.03 ^a^	0.18 ± 0.01 ^a^	0.17 ^I^
	8	0.09 ± 0.00 ^b^	0.01 ± 0.00 ^d^	0.10 ± 0.00 ^b^	0.01 ^II^
	16	0.07 ± 0.00 ^c^	0.08 ± 0.00 ^c^	0.01 ± 0.00 ^d^	0.08 ^III^
	Average	0.10 ^B^	0.11 ^B^	0.13 ^A^	
Urease (Ure), mM N-NH_4_	0	0.74 ± 0.06 ^c^	0.94 ± 0.03 ^a^	0.80 ± 0.01 ^b^	0.83 ^I^
	8	0.15 ± 0.05 ^ef^	0.26 ± 0.00 ^d^	0.80 ± 0.03 ^bc^	0.40 ^II^
	16	0.10 ± 0.00 ^f^	0.18 ± 0.01 ^e^	0.18 ± 0.03 ^e^	0.15 ^III^
	Average	0.33 ^C^	0.46 ^B^	0.59 ^A^	
Acid phosphatase (AcP), mM PNP	0	2.60 ± 0.00 ^b^	2.86 ± 0.02 ^a^	1.88 ± 0.20 ^e^	2.45 ^I^
	8	2.01 ± 0.02 ^de^	2.25 ± 0.01 ^c^	2.14 ± 0.15 ^cd^	2.13 ^II^
	16	1.53 ± 0.00 ^f^	1.99 ± 0.00 ^de^	2.09 ± 0.00 ^d^	1.87 ^III^
	Average	2.05 ^B^	2.37 ^A^	2.04 ^B^	
Alkaline phosphatase (AlP), mM PNP	0	0.48 ± 0.01 ^c^	0.53 ± 0.01 ^b^	0.72 ± 0.01 ^a^	0.58 ^I^
	8	0.34 ± 0.00 ^ef^	0.36 ± 0.02 ^e^	0.43 ± 0.00 ^d^	0.38 ^II^
	16	0.26 ± 0.01 ^h^	0.29 ± 0.02 ^g^	0.34 ± 0.00 ^f^	0.30 ^III^
	Average	0.36 ^C^	0.39 ^B^	0.49 ^A^	
*β*-glucosidase (Glu), mM PNP kg^−1^	0	0.24 ± 0.01 ^b^	0.25 ± 0.00 ^ab^	0.26 ± 0.01 ^a^	0.25 ^I^
	8	0.19 ± 0.01 ^d^	0.22 ± 0.01 ^c^	0.22 ± 0.00 ^c^	0.21 ^II^
	16	0.16 ± 0.00 ^e^	0.19 ± 0.00 ^d^	0.19 ± 0.00 ^d^	0.18 ^III^
	Average	0.20 ^B^	0.22 ^A^	0.22 ^A^	
Arylsulfatase (Aryl), mM PNS kg^−1^	0	0.10 ± 0.00 ^d^	0.12 ± 0.01 ^b^	0.13 ± 0.00 ^a^	0.12 ^I^
	8	0.09 ± 0.09 ^e^	0.11 ± 0.00 ^c^	0.12 ± 0.01 ^b^	0.12 ^I^
	16	0.06 ± 0.00 ^f^	0.10 ± 0.00 ^d^	0.12 ± 0.01 ^b^	0.09 ^II^
	Average	0.09 ^C^	0.11 ^B^	0.12 ^A^	

The same letters (a–h) within each property indicate a homogeneous group, A–C for the sorbent, I–III for the dose of the petroleum product, *p* < 0.050, N for each standard deviation = 4, and N for each property tested = 36.

**Table 3:** Physicochemical properties of gasoline-contaminated soil (G).

Properties	Dose Gasoline (G), cm^3^ kg^−1^ d.m. of Soil	Sorbent (S)			Average
		Control (C)	Vermiculite (V)	Agrobasalt (A)	
Total organic carbon (C_org_) in g kg^−1^	0	7.3 ± 0.1 ^b^	7.9 ± 0.1 ^a^	7.3 ± 0.1 ^b^	7.5 ^I^
	8	7.1 ± 0.1 ^c^	7.0 ± 0.0 ^c^	7.1 ± 0.0 ^c^	7.1 ^II^
	16	6.8 ± 0.01 ^d^	6.8 ± 0.1 ^d^	7.1 ± 0.0 ^c^	6.9 ^III^
	Average	7.1 ^C^	7.3 ^A^	7.2 ^B^	
Total nitrogen (N_Total_) in g kg^−1^	0	1.20 ± 0.03 ^b^	1.16 ± 0.00 ^c^	1.12 ± 0.00 ^d^	1.16 ^II^
	8	1.15 ± 0.00 ^c^	1.11 ± 0.02 ^d^	1.09 ± 0.00 ^e^	1.12 ^III^
	16	1.26 ± 0.01 ^a^	1.19 ± 0.00 ^b^	1.14 ± 0.01 ^c^	1.20 ^I^
	Average	1.21 ^A^	1.15 ^B^	1.12 ^C^	
C:N	0	6.0	6.8	6.5	6.5
	8	6.2	6.3	6.5	6.3
	16	5.4	5.7	6.2	5.8
	Average	5.9	6.3	6.4	
pH_KCl_	0	4.3 ± 0.1 ^c^	4.3 ± 0.0 ^c^	4.4 ± 0.1 ^b^	4.3 ^II^
	8	4.3 ± 0.0 ^c^	4.3 ± 0.0 ^c^	4.4 ± 0.0 ^b^	4.3 ^II^
	16	4.3 ± 0.0 ^c^	4.4 ± 0.0 ^b^	4.5 ± 0.0 ^a^	4.4 ^I^
	Average	4.3 ^B^	4.3 ^B^	4.4 ^A^	
Hydrolytic acidity (HAC) in mmol^(+)^ kg^−1^ soil	0	33.4 ± 0.4 ^cd^	35.3 ± 0.0 ^b^	36.0 ± 0.8 ^a^	34.9 ^I^
	8	35.6 ± 0.0 ^b^	34.5 ± 0.0 ^c^	33.4 ± 0.4 ^cd^	34.4 ^II^
	16	35.6 ± 0.4 ^b^	33.8 ± 0.0 ^cd^	33.0 ± 0.0 ^d^	34.1 ^II^
	Average	34.8 ^A^	34.5 ^A^	34.1 ^B^	
Total exchangeable base cations (EBC) in mmol^(+)^ kg^−1^ soil	0	44.8 ± 2.1 ^e^	49.2 ± 0.0 ^cd^	52.3 ± 1.0 ^bc^	48.7 ^I^
	8	43.9 ± 1.1 ^e^	47.2 ± 0.0 ^d^	54.3 ± 3.1 ^ab^	48.5 ^I^
	16	43.7 ± 1.2 ^e^	49.2 ± 4.1 ^cd^	56.4 ± 1.0 ^a^	49.8 ^I^
	Average	44.1 ^C^	48.5 ^B^	54.3 ^A^	
Total cation exchange capacity of soil (CEC) in mmol^(+)^ kg^−1^ soil	0	78.1 ± 1.8 ^e^	84.5 ± 0.0 ^bc^	88.3 ± 1.8 ^ab^	83.6 ^I^
	8	79.2± 1.1 ^de^	81.7 ± 0.0 ^d^	87.7 ± 2.7 ^ab^	82.8 ^I^
	16	79.4 ± 1.5 ^de^	83.0 ± 4.1 ^cd^	89.4 ± 1.0 ^a^	83.9 ^I^
	Average	78.9 ^C^	83.0 ^B^	88.5 ^A^	
Base cations saturation ratio in soil (BS) in %	0	55.9 ± 1.4 ^bc^	56.8 ± 0.0 ^b^	57.8 ± 0.0 ^b^	56.8 ^II^
	8	54.1 ± 0.6 ^cd^	56.3 ± 0.0 ^b^	60.4 ± 1.6 ^a^	56.9 ^II^
	16	53.8 ± 0.5 ^d^	57.8 ± 2.0 ^b^	61.5 ± 04 ^a^	57.7 ^I^
	Average	54.6 ^C^	57.0 ^B^	59.9 ^A^	
Electrical conductivity of soils (EC), μS cm^−1^	0	39.3 ± 0.6 ^g^	44.7 ± 2.0 ^f^	47.6 ± 0.8 ^f^	43.9 ^III^
	8	65.7 ± 1.9 ^d^	68.9 ± 1.4 ^d^	58.9 ± 2.1 ^e^	64.5 ^II^
	16	115.0 ± 1.9 ^a^	93.4 ± 2.1 ^b^	87.7 ± 2.7 ^c^	98.7 ^I^
	Average	73.3 ^A^	69.0 ^B^	64.7 ^C^	
Specific density, g cm^−3^	0	2.55 ± 0.13 ^a^	2.58 ± 0.13 ^a^	2.51 ± 0.12 ^a^	2.54 ^I^
	8	2.58 ± 0.15 ^a^	2.52 ± 0.10 ^a^	2.57 ± 0.13 ^a^	2.56 ^I^
	16	2.56 ± 0.12 ^a^	2.50 ± 0.13 ^a^	2.57 ± 0.14 ^a^	2.54 ^I^
	Average	2.56 ^A^	2.53 ^A^	2.55 ^A^	
Redox potential (Eh), mV	0	445.6 ± 11.1 ^a^	439.6 ± 8.1 ^a^	443.0 ± 8.9 ^a^	442.7 ^I^
	8	444.6 ± 9.3 ^a^	438.2 ± 8.8 ^a^	429.9 ± 8.6 ^a^	437.6 ^I^
	16	428.7 ± 8.6 ^a^	430.4 ± 10.8 ^a^	428.4 ± 8.6 ^a^	429.2 ^II^
	Average	439.6 ^A^	436.0 ^A^	433.8 ^A^	

The same letters (a–g) within each property indicate a homogeneous group, A–C for the sorbent, I–III for the dose of the petroleum product, *p* < 0.050, N for each standard deviation = 4, and N for each property tested = 36.

**Table 4:** *Zea mays* yield and leaf greenness index (SPAD) in soil contaminated with diesel oil (DO) (g d.m. of pot^−1^).

Properties	Dose Diesel Oil (DO), cm^3^ kg^−1^ d.m. of Soil	Sorbent (S)			Average
		Control (C)	Vermiculite (V)	Agrobasalt (A)	
Aerial parts (Ya), g d.m. pot^−1^	0	74.6 ± 0.4 ^a^	71.4 ± 1.9 ^b^	67.8 ± 1.7 ^c^	71.3 ^I^
	8	19.1 ± 0.6 ^e^	24.8 ± 1.5 ^d^	23.6 ± 0.5 ^d^	22.5 ^II^
	16	8.0 ± 0.3 ^g^	10.6 ± 0.0 ^f^	10.4 ± 0.1 ^f^	9.7 ^III^
	Average	33.9 ^B^	35.6 ^A^	33.9 ^B^	
Root yield (Yr), g d.m. pot^−1^	0	14.6 ± 0.8 ^a^	11.4 ± 0.9 ^b^	8.4 ± 0.8 ^c^	11.5 ^I^
	8	3.4 ± 0.1 ^e^	4.0 ± 0.3 ^de^	5.0 ± 0.4 ^d^	4.1 ^II^
	16	2.1 ± 0.3 ^f^	3.2 ± 0.3 ^e^	2.5 ± 0.3 ^f^	2.6 ^III^
	Average	6.7 ^A^	6.2 ^A^	5.3 ^B^	
Leaf greenness index (SPAD)	0	40.6 ± 0.8 ^b^	42.2 ± 0.3 ^a^	42.4 ± 0.5 ^a^	41.7 ^I^
	8	40.4 ± 0.6 ^b^	40.8 ± 0.8 ^ab^	42. 8 ± 0.5 ^a^	41.3 ^I^
	16	37.6 ± 0.5 ^c^	36.8 ± 0.4 ^d^	33.6 ± 0.2 ^e^	36.0 ^II^
	Average	39.5 ^A^	39.9 ^A^	39.6 ^A^	

The same letters (a–g) within each property indicate a homogeneous group, A–B for the sorbent, I–III for the dose of the petroleum product, *p* < 0.050, N for each standard deviation = 4, and N for each property tested = 3.

**Table 5:** Enzyme activity in 1 kg of soil contaminated with diesel oil (DO) in 1 h.

Enzyme	Dose Diesel Oil (DO), cm^3^ kg^−1^ d.m. of Soil	Sorbent (S)			Average
		Control (C)	Vermiculite (V)	Agrobasalt (A)	
Dehydrogenases (Deh), mM TFF	0	12.00 ± 0.26 ^d^	16.04 ± 0.11 ^a^	14.51 ± 0.11 ^bc^	14.19 ^III^
	8	13.85 ± 0.03 ^c^	16.23 ± 0.24 ^a^	14.89 ± 0.56 ^b^	14.99 ^II^
	16	14.79 ± 0.28 ^b^	16.75 ± 0.67 ^a^	15.08 ± 063 ^b^	15.54 ^I^
	Average	13.55 ^C^	16.34 ^A^	14.82 ^B^	
Catalase (Cat), M O_2_	0	0.16 ± 0.00 ^d^	0.16 ± 0.03 ^d^	0.18 ± 0.01 ^d^	0.17 ^III^
	8	0.34 ± 0.01 ^c^	0.40 ± 0.00 ^b^	0.44 ± 0.00 ^a^	0.39 ^II^
	16	0.43 ± 0.00 ^a^	0.40 ± 0.01 ^b^	0.44 ± 0.00 ^a^	0.42 ^I^
	Average	0.31 ^B^	0.32 ^B^	0.35 ^A^	
Urease (Ure), mM N-NH_4_	0	0.74 ± 0.06 ^d^	0.94 ± 0.03 ^a^	0.80 ± 0.01 ^bcd^	0.83 ^I^
	8	0.77 ± 0.05 ^cd^	0.86 ± 0.01 ^b^	0.61 ± 0.04 ^e^	0.75 ^II^
	16	0.78 ± 0.04 ^cd^	0.84 ± 0.03 ^bc^	0.59 ± 0.03 ^e^	0.74 ^II^
	Average	0.76 ^B^	0.88 ^A^	0.67 ^C^	
Acid phosphatase (AcP), mM PNP	0	2.60 ± 0.00 ^b^	2.86 ± 0.02 ^a^	1.88 ± 0.20 ^e^	2.45 ^I^
	8	2.08 ± 0.00 ^d^	2.48 ± 0.14 ^b^	2.24 ± 0.02 ^c^	2.27 ^II^
	16	2.09 ± 0.01 ^d^	2.52 ± 0.03 ^b^	2.13 ± 0.01 ^cd^	2.25 ^II^
	Average	2.26 ^B^	2.62 ^A^	2.08 ^C^	
Alkaline phosphatase (AlP), mM PNP	0	0.48 ± 0.01 ^i^	0.53 ± 0.01 ^h^	0.72 ± 0.01 ^f^	0.58 ^III^
	8	0.98 ± 0.01 ^d^	0.75 ± 0.00 ^e^	1.18 ± 0.02 ^c^	0.97 ^II^
	16	1.44 ± 0.01 ^a^	0.67 ± 0.01 ^g^	1.29 ± 0.01 ^b^	1.14 ^I^
	Average	0.97 ^B^	0.65 ^C^	1.07 ^A^	
*β*-glucosidase (Glu), mM PNP kg^−1^	0	0.24 ± 0.00 ^e^	0.25 ± 0.00 ^d^	0.26 ± 0.00 ^c^	0.25 ^II^
	8	0.26 ± 0.01 ^c^	0.27 ± 0.00 ^b^	0.27 ± 0.00 ^b^	0.27 ^I^
	16	0.27 ± 0.00 ^b^	0.28 ± 0.00 ^a^	0.29 ± 0.00 ^a^	0.28 ^I^
	Average	0.26 ^B^	0.27 ^A^	0.27 ^A^	
Arylsulfatase (Aryl), mM PNS kg^−1^	0	0.10 ± 0.00 ^e^	0.12 ± 0.01 ^d^	0.13 ± 0.00 ^d^	0.12 ^III^
	8	0.15 ± 0.01 ^cd^	0.15 ± 0.01 ^cd^	0.16 ± 0.01 ^c^	0.15 ^II^
	16	0.15 ± 0.01 ^cd^	0.17 ± 0.01 ^b^	0.21 ± 0.00 ^a^	0.18 ^I^
	Average	0.13 ^C^	0.15 ^B^	0.16 ^A^	

The same letters (a–i) within each property indicate a homogeneous group, A–C for the sorbent, I–III for the dose of the petroleum product, *p* < 0.050, N for each standard deviation = 4, and N for each property tested = 36.

**Table 6:** Physicochemical properties of soil contaminated with diesel oil (DO).

Properties	Dose Diesel Oil (DO), cm^3^ kg^−1^ d.m. of Soil	Sorbent (S)			Average
		Control (C)	Vermiculite (V)	Agrobasalt (A)	
Total organic carbon (C_org_) in g kg^−1^	0	7.3 ± 0.1 ^g^	7.9 ± 0.1 ^f^	7.3 ± 0.1 ^g^	7.5 ^III^
	8	8.6 ± 0.0 ^e^	8.9 ± 0.0 ^d^	8.7 ± 0.0 ^e^	8.7 ^II^
	16	10.1 ± 0.0 ^b^	10.3 ± 0.2 ^c^	11.2 ± 0.0 ^a^	10.5 ^I^
	Average	8.6 ^B^	9.0 ^A^	9.1 ^A^	
Total nitrogen (N_Total_) in g kg^−1^	0	1.20 ± 0.02 ^e^	1.16 ± 0.00 ^f^	1.12 ± 0.00 ^g^	1.16 ^III^
	8	1.21 ± 0.01 ^de^	1.22 ± 0.01 ^de^	1.23 ± 0.01 ^d^	1.22 ^II^
	16	1.36 ± 0.00 ^a^	1.32 ± 0.02 ^b^	1.27 ± 0.01 ^c^	1.32 ^I^
	Average	1.26 ^A^	1.23 ^B^	1.21 ^C^	
C:N	0	6.0	6.8	6.5	6.5
	8	7.1	7.3	7.0	7.1
	16	7.4	7.8	8.8	8.0
	Average	6.8	7.3	7.4	
pH_KCl_	0	4.3 ± 0.1 ^f^	4.3 ± 0.0 ^f^	4.4 ± 0.1 ^f^	4.3 ^III^
	8	4.6 ± 0.0 ^e^	5.0 ± 0.0 ^b^	4.7 ± 0.1 ^d^	4.8 ^II^
	16	4.9 ± 0.1 ^c^	4.8 ± 0.0 ^c^	5.1 ± 0.0 ^a^	4.9 ^I^
	Average	4.6 ^B^	4.7 ^A^	4.7 ^A^	
Hydrolytic acidity (HAC) in mM^(+)^ kg^−1^ soil	0	33.4 ± 0.4 ^b^	35.3 ± 0.0 ^a^	36.0 ± 0.8 ^a^	34.9 ^I^
	8	30.0 ± 0.0 ^c^	32.6 ± 0.0 ^b^	30.4 ± 0.4 ^c^	31.0 ^II^
	16	27.4 ± 0.4 ^e^	28.9 ± 0.4 ^d^	26.6 ± 0.4 ^e^	27.6 ^III^
	Average	30.3 ^C^	32.3 ^A^	31.0 ^B^	
Total exchangeable base cations (EBC) in mM^(+)^ kg^−1^ soil	0	44.8 ± 2.1 ^f^	49.2 ± 0.0 ^de^	52.3 ± 1.0 ^cd^	48.7 ^III^
	8	47.5 ± 2.1 ^e^	49.2 ± 2.1 ^de^	64.6 ± 1.0 ^a^	53.8 ^II^
	16	55.4 ± 3.6 ^bc^	57.4 ± 4.1 ^b^	67.7 ± 2.1 ^a^	60.1 ^I^
	Average	49.2 ^C^	51.9 ^B^	61.5 ^A^	
Total cation exchange capacity of soil (CEC) in mM^(+)^ kg^−1^ soil	0	78.1 ± 1.8 ^ef^	84.5 ± 0.0 ^bc^	88.3 ± 1.8 ^b^	83.6 ^II^
	8	77.5 ± 2.1 ^f^	81.8 ± 2.4 ^cd^	95.0± 1.4 ^a^	84.8 ^II^
	16	82.7 ± 3.9 ^cd^	86.3 ± 3.7 ^bc^	94.3 ± 1.7 ^a^	87.8 ^I^
	Average	79.5 ^C^	84.2 ^B^	92.5 ^A^	
Base cations saturation ratio in soil (BS) in %	0	55.9 ± 1.4 ^e^	56.8 ± 0.0 ^de^	57.8 ± 0.0 ^de^	56.8 ^III^
	8	59.8 ± 1.1 ^c^	58.6 ± 0.7 ^cd^	66.4 ± 0.1 ^b^	61.6 ^II^
	16	65.2 ± 1.2 ^b^	64.9 ± 1.8 ^b^	69.1 ± 0.9 ^a^	66.7 ^I^
	Average	60.3 ^B^	60.1 ^B^	64.7 ^A^	
Electrical conductivity of soils (EC), μS cm^−1^	0	39.3 ± 0.6 ^g^	44.7 ± 2.0 ^f^	47.6 ± 0.8 ^f^	43.9 ^III^
	8	89.5 ± 1.2 ^d^	105.1 ± 0.9 ^b^	77.5 ± 1.4 ^e^	90.7 ^II^
	16	99.4 ± 2.8 ^c^	134.1 ± 1.0 ^a^	136.7 ± 1.1 ^a^	123.4 ^I^
	Average	76.1 ^C^	94.6 ^A^	87.3 ^B^	
Specific density, g cm^−3^	0	2.55 ± 0.13 ^a^	2.58 ± 0.13 ^a^	2.51 ± 0.12 ^a^	2.54 ^I^
	8	2.45 ± 0.12 ^a^	2.41 ± 0.12 ^a^	2.46 ± 0.12 ^a^	2.44 ^I^
	16	2.43 ± 0.12 ^a^	2.40 ± 0.12 ^a^	2.46 ± 0.12 ^a^	2.43 ^I^
	Average	2.48 ^A^	2.46 ^A^	2.48 ^A^	
Redox potential (Eh), mV	0	445.6 ± 11.1 ^a^	439.6 ± 8.1 ^ab^	443.0 ± 8.9 ^ab^	442.7 ^I^
	8	406.2 ± 8.1 ^cd^	428.3 ± 6.4 ^abc^	423.2 ± 10.6 ^abc^	419.2 ^II^
	16	386.0 ± 7.7 ^d^	410.1 ± 8.6 ^cd^	419.9 ± 8.4 ^bc^	405.3 ^III^
	Average	412.6 ^B^	426.0 ^A^	428.7 ^A^	

The same letters (a–g) within each property indicate a homogeneous group, A–C for the sorbent, I–III for the dose of the petroleum product, *p* < 0.050, N for each standard deviation = 4, and N for each property tested = 36.

**Table 7:** Scheme for the determination of soil enzyme activity.

C-cycle
Dehydrogenases (Deh): 1 g soil + 2,3,5–triphenyl tetrazolium chloride (substrate) → triphenyl fomazan − TFF (product) [µM TFF kg^−1^ h^−1^] Catalase (Cat): 2 g soil + H_2_O_2_ − aqueus solution (substrate) → O_2_ (product) [M O_2_ kg^−1^ h^−1^] *ß*-glucosidase (Glu): 1 g soil + 4–nitrophenyl–*ß*–D–glucopyranoside (substrate) → 4–nitrophenol − PN (product) [mM PN kg^−1^ h^−1^]
N-cycle
Urease (Ure): 10 g soil + urea – aqueous solution (substrate) → N-NH_4_ (product) [mM N-NH_4_ kg^−1^ h^−1^]
P-cycle
Acid phosphatase (AcP): 1 g soil + disodium 4–nitrophenyl phosphate hexahydrate (substrate) → 4-nitro-phenol − PN (product) [mM PN kg^−1^ h^−1^] Alkaline phosphatase (AlP): 1 g soil + disodium 4–nitrophenyl phosphate hexahydrate (substrate) → 4-nitrophenol − PN (product) [mM PN kg^−1^ h^−1^]
S-cycle
Aryosulfatase (Aryl): 1 g soil + potassium–4–nitrophenyl-sulfate (substrate) → 4-nitrophenol − PN (product) [mM PN kg^−1^ h^−1^]

## Data Availability

The data presented in this study are available on request from the corresponding author.

## Supplementary Materials

The following supporting information can be downloaded at: https://www.mdpi.com/article/10.3390/molecules29245838/s1, Table S1: Simple correlation coefficients in diesel and petrol contaminated soil.
