# Supplementary material for: Revitalization of Soil Contaminated by Petroleum Products Using Materials That Improve the Physicochemical and Biochemical Properties of the Soil
Source: Molecules. 2024 Dec 11;29(24):5838. doi: 10.3390/molecules29245838 (PMC11677455; doi:10.3390/molecules29245838)
Supplement: Supplementary file 1 [file molecules-29-05838-s001.zip › molecules-3256245-supplementary.pdf]

Supplementary Material

Revitalization of soil contaminated by petroleum products using materials that improve the physicochemical and biochemical properties of the soil

Jadwiga Wyszkowska *, Agata Borowik, Magdalena Zaborowska and Jan Kucharski

Department of Soil Science and Microbiology, Faculty of Agriculture and Forestry, University of Warmia and Mazury in Olsztyn, 10-719 Olsztyn, Poland; agata.borowik@uwm.edu.pl; m.zaborowska@uwm.edu.pl; jan.kucharski@uwm.edu.pl
* Correspondence jadwiga.wyszkowska@uwm.edu.pl

Table S1. Simple correlation coefficients in diesel and petrol contaminated soil

		Ya	Yr	SPAD	Deh	Cat	Ure	AcP	AlP	Aryl	Glu	C_{org}	N_{Total}	pH	HAC	EBC	CEC	BS	BA	
Ya		1.000	0.850*	−0.800*	0.932*	0.722*	0.755*	0.752*	0.696*	0.699*	0.894*	0.605*	−0.604*	−0.280	−0.107	0.139	0.122	0.171	0.938*	
Yr		0.945*	1.000	−0.911*	0.783*	0.818*	0.800*	0.803*	0.668*	0.460*	0.842*	0.695*	−0.239	−0.469*	−0.091	−0.041	−0.067	0.007	0.814*	
SPAD		0.618*	0.518*	1.000	−0.775*	−0.794*	−0.842*	−0.621*	−0.738*	−0.434*	−0.872*	−0.614*	0.251	0.518*	−0.097	0.055	0.035	0.058	−0.798*	
Deh		−0.393*	−0.485*	−0.186	1.000	0.760*	0.798*	0.683*	0.783*	0.690*	0.903*	0.747*	−0.634*	−0.299	0.154	0.135	0.184	0.093	0.997*	
Cat		−0.960*	−0.896*	−0.522*	0.399*	1.000	0.841*	0.543*	0.919*	0.589*	0.892*	0.748*	−0.243	−0.292	0.193	0.157	0.217	0.098	0.787*	
Ure		0.351*	0.256	0.334*	0.366*	−0.444*	1.000	0.611*	0.826*	0.625*	0.880*	0.736*	−0.348*	−0.222	0.055	0.278	0.312	0.240	0.831*	
AcP		0.353*	0.507*	0.150	0.213	−0.324	0.475*	1.000	0.344*	0.476*	0.643*	0.745*	−0.262*	−0.249	−0.244	0.026	−0.034	0.110	0.718*	
AlP		−0.740*	−0.741*	−0.444*	0.040	0.767*	−0.610*	−0.617*	1.000	0.660*	0.885*	0.640*	−0.399*	−0.171	0.329*	0.274	0.378*	0.159	0.794*	
Aryl	Diesel oil	−0.825*	−0.818*	−0.732*	0.462*	0.824*	−0.493*	−0.364*	0.678*	1.000	0.692*	0.497*	−0.742*	0.362*	−0.207	0.698*	0.696*	0.674*	0.699*	Gasoline
Glu		−0.849*	−0.856*	−0.652*	0.533*	0.872*	−0.397*	−0.383*	0.704*	0.899*	1.000	0.710*	−0.491*	−0.309	0.126	0.182	0.227	0.145	0.919*	
C_{org}		−0.862*	−0.793*	−0.870*	0.470*	0.818*	−0.334*	−0.176	0.649*	0.896*	0.892*	1.000	−0.254	−0.245	0.234	0.129	0.198	0.058	0.773*	
N_{Total}		−0.807*	−0.658*	−0.705*	0.251	0.754*	−0.208	−0.090	0.603*	0.581*	0.667*	0.810*	1.000	−0.336*	0.165	−0.542*	−0.539*	−0.519*	−0.607*	
pH		−0.883*	−0.857*	−0.663*	0.479*	0.909*	−0.348*	−0.257	0.657*	0.883*	0.863*	0.869*	0.678*	1.000	−0.432*	0.782*	0.730*	0.779*	−0.297	
HAC		0.894*	0.760*	0.780*	−0.129	−0.845*	0.529*	0.298	−0.775*	−0.775*	−0.774*	−0.894*	−0.866*	−0.763*	1.000	−0.396*	−0.171	−0.615*	0.130	
EBC		−0.563*	−0.544*	−0.491*	0.326	0.659*	−0.664*	−0.333*	0.657*	0.786*	0.784*	0.686*	0.407*	0.606*	−0.610	1.000	0.972*	0.967*	0.147	
CEC		−0.231	−0.276	−0.201	0.334*	0.374*	−0.543*	−0.255	0.407*	0.566*	0.564*	0.382*	0.054	0.351*	−0.235	0.913*	1.000	0.881*	0.191	
BS		−0.793*	−0.727*	−0.677*	0.297	0.826*	−0.646*	−0.368*	0.793*	0.871*	0.877*	0.866*	0.683*	0.750*	−0.860*	0.926*	0.695*	1.000	0.111	
BA		−0.493*	−0.546*	−0.257	0.981*	0.506*	0.306	0.247	0.155	0.522*	0.594*	0.567*	0.381*	0.567*	−0.260	0.377*	0.328	0.389*	1.000	

Explanations of abbreviations are presented in Table 1, 2 and 3, *- statistically significant correlation coefficients, $p < 0.050$, N = 36.
